# Effect of Ginkgo Leaf Parenteral Solution on Blood and Cochlea Antioxidant and Immunity Indexes in OM Rats

**DOI:** 10.3390/molecules161210433

**Published:** 2011-12-15

**Authors:** Jiandong Zhao, Yu Su, Aiting Chen, Hu Yuan, Liangfa Liu, Wenming Wu

**Affiliations:** Department of Otolaryngology/Head and Neck Surgery, PLA General Hospital, Beijing 100853, China

**Keywords:** otitis media, rat, ginkgo leaf parenteral solution, antioxidant, immunity

## Abstract

Oxidative stress is involved in the development and progression of otitis media (OM). In this study, we investigated the effect of Ginkgo leaf parenteral solution on blood and cochlea antioxidant and immunity indexs in OM rats. In OM model rats, blood and cochlea malondialdehyde (MDA), nitric oxide (NO), tumor necrosis factor-alpha (TNF-α), interleukin-1 beta (IL-1β), interleukin-6 (IL-6), interleukin-8 (IL-8) and interleukin-10 (IL-10) levels were significantly increased, whereas antioxidant enzymes activities (superoxide dismutase (SOD), catalase (CAT), glutathione peroxidase (GSH-Px) and glutathione reductase (GR)) were significantly decreased compared with normal rats. Treatment with Ginkgo leaf parenteral solution restored the altered parameters in a dose-dependent manner. We conclude that Ginkgo leaf parenteral solution confers protection against oxidative injuries in OM rats by increasing activities of antioxidants and immunity, suggesting a potential drug for the prevention and therapy of OM.

## 1. Introduction

Acute otitis media (AOM) occurring early in infancy may be an important predictor of later morbidity, including psychological and educational difficulties. Additionally, AOM causes much suffering. Concern about such morbidity, both immediate and long-term, has lead to a search for effective strategies to prevent this illness. Since free radicals were first identified in the early 1900s, they have been implicated in the pathogenesis of a number of diseases with an inflammatory component, including rheumatoid arthritis, atherosclerosis [1], pulmonary emphysema, inflammatory bowel disease, and periodontal disease [2]. They are also believed to play a role in stroke, e.g., myocardial infarction [3], and cataracts [4], as well as the degenerative processes associated with aging [5]. Radiation, oxygen toxicity, ischemia-reperfusion damage, infections and inflammation are some of the causes of increased FORs production [6,7]. The increased production of free oxygen radicals (FORs) in inflammation is due to the presence of leukocytes in the affected area [8]. Recent work has examined the role of free radicals in acute otitis media [9].

Ginkgo (*Ginkgo biloba*) is one of the most popular herbal medicines, and extracts from its leaves have been used as food supplements or health foods without any restriction in Japan as well as the United States. Additionally, in many European countries these extracts have been used clinically. The Ginkgo leaf contains many bioactive compounds, including flavonol and flavone glycosides, terpenoids and ginkgolides [10]. Many studies have reported several physiological actions attributed to these compounds, including antioxidant and vasoactivating properties, inhibition of platelet activating factor and neuro-transmitter modulation [11,12,13,14,15,16,17,18]. In the literature, several studies have concentrated on the role of free oxygen radicals and nitric oxide in the development of myringosclerosis in traumatized tympanic membranes and otitis media with effusion [19,20,21]. The present study was performed to investigate the effect of Ginkgo leaf parenteral solution on nitric oxide, antioxidant enzymes and immunity indexes in rats with otitis media.

## 2. Results

Table 1 shows the content of MDA and NO in tissues of normal and experimental groups. There was a significant increase in the concentration of MDA and NO in tissues of OM rats (Group II) compared to the corresponding control group (I). Administration of Ginkgo leaf parenteral solution dose-dependently significantly decreased the content of MDA and NO in the blood and cochlea of OM rats (Groups III and IV). 

**Table 1:** Effect of Ginkgo leaf parenteral solution on the content of MDA and reduced glutathione (GSH) in tissues of normal and experimental groups.

Group	MDA		NO (nmol/mg protein)	
	Serum (nmol/mL)	Cochlea (nmol/mg protein)	Serum (μmol/L)	Cochlea (μmol/g tissue)
I	3.85 ± 0.14	2.17 ± 0.11	127.37 ± 7.04	82.49 ± 7.93
II	7.58 ± 0.32 **	8.64 ± 0.35 **	253.32 ± 18.61 **	121.33 ± 8.71 **
III	5.07 ± 0.21 ^##^	4.62 ± 0.19 ^##^	199.54 ± 10.12 ^##^	100.39 ± 9.01 ^##^
IV	4.11 ± 0.19 ^##^	2.98 ± 0.14 ^##^	136.14 ± 11.53 ^##^	90.44 ± 7.13 ^##^
V	3.88 ± 0.42 ^##^	2.31 ± 0.18 ^##^	130.5 ± 12.54 ^##^	84.51 ± 9.04 ^##^

** *p* < 0.01, compared with group I; ^##^*p* < 0.01, compared with group II.

Table 2 shows the levels of enzymic antioxidants (SOD, CAT) in the blood and cochlea of control and experimental rats. The activities of SOD, CAT were found to be significantly decreased in the blood and cochlea of OM rats (Group II) when compared to normal rats (Group I). Administration of Ginkgo leaf parenteral solution for 15 days dose-dependently significantly enhanced the SOD, CAT activities in Group III and IV rats when compared to Group II rats.

**Table 2:** Effect of Ginkgo leaf parenteral solution on enzymic antioxidants (SOD, CAT) in the blood and cochlea of control and experimental rats.

Group	SOD		CAT	
	Serum (U/mL)	Cochlea (U/mg protein)	Serum (U/mL)	Cochlea (U/mg protein)
I	254.1 ± 12.74	291.3 ± 12.43	102.64 ± 6.42	53.07 ± 2.18
II	121.9 ± 6.03 **	133.8 ± 11.21 **	35.49 ± 1.92 **	22.16 ± 1.05 **
III	184.8 ± 7.29 ^##^	196.4 ± 12.41 ^##^	57.21 ± 2.06 ^##^	34.61 ± 1.74 ^##^
IV	231.7 ± 11.05 ^##^	277.5 ± 18.39 ^##^	92.17 ± 3.94 ^##^	49.52 ± 1.77 ^##^
V	257.1 ± 15.9 ^##^	300.5 ± 23.84 ^##^	118.5 ± 9.04 ^##^	52.73 ± 3.04 ^##^

** *p* < 0.01, compared with group I; ^##^*p* < 0.01, compared with group II.

Table 3 shows the levels of enzymic antioxidants (GSH-Px, GR) in the blood and cochlea of control and experimental rats. The activity of GSH-Px and GR enzymes also showed similar pattern to that of SOD and CAT. The activities of GSH-Px, GR were found to be significantly decreased in the blood and cochlea of OM rats (Group II) when compared to normal rats (group I). Administration of Ginkgo leaf parenteral solution for 15 days dose-dependently significantly enhanced the GSH-Px, GR activities in Group III and IV rats when compared to Group II rats. 

**Table 3:** Effect of Ginkgo leaf parenteral solution on enzymic antioxidants (GSH-Px, GR) in the blood and cochlea of control and experimental rats.

Group	GSH-Px		GR	
	Serum (U/mL)	Cochlea (U/mg protein)	Serum (U/mL)	Cochlea (U/mg protein)
I	48.82 ± 1.74	84.37 ± 6.09	21.18 ± 1.21	44.17 ± 1.88
II	18.52 ± 1.47 **	20.07 ± 1.67 **	10.37 ± 1.01 **	20.14 ± 1.53 **
III	32.74 ± 1.33 ^##^	51.02 ± 3.83 ^##^	15.66 ± 1.12 ^##^	35.29 ± 1.52 ^##^
IV	46.71 ± 2.07 ^##^	70.75 ± 4.81 ^##^	24.18 ± 0.08 ^##^	40.81 ± 1.47 ^##^
V	49.11 ± 3.21 ^##^	80.53 ± 7.02 ^##^	30.14 ± 1.53 ^##^	45.29 ± 2.74 ^##^

** *p* < 0.01, compared with group I; ^##^*p* < 0.01, compared with group II.

Table 4 summarizes the change of immunity indexs in cochlea of rats. The TNF-α, IL-1β, IL-6, IL-8 and IL-10 levels were significantly increased in group II when compared with group I. However, administration of Ginkgo leaf parenteral solution for 15 days dose-dependently significantly decreased the TNF-α, IL-1β, IL-6, IL-8 and enhanced IL-10 levels in cochlea of Group III and IV rats when compared with the OM model group (II).

**Table 4:** Effect of Ginkgo leaf parenteral solution on immunity indexs in cochlea of rats.

Group	TNF-α (ng/mL)	IL-1β (ng/L)	IL-6 (ng/L)	IL-8 (ng/L)	IL-10 (ng/L)
I	2.52 ± 0.13	9.04 ± 0.52	69.52 ± 1.86	75.28 ± 3.11	6.07 ± 3.02
II	6.51 ± 0.32 **	21.63 ± 1.04 **	116.3 ± 5.82 **	177.42 ± 7.93 **	26.87 ± 1.52 **
III	4.83 ± 0.22 ^##^	16.44 ± 0.89 ^##^	89.74 ± 2.79 ^##^	142.14 ± 8.83 ^##^	30.51 ± 1.68 ^##^
IV	2.99 ± 0.11 ^##^	11.43 ± 0.08 ^##^	72.18 ± 4.16 ^##^	105.62 ± 7.27 ^##^	34.17 ± 1.92 ^##^
V	2.28 ± 0.09 ^##^	10.27 ± 1.32 ^##^	68.16 ± 3.81 ^##^	84.29 ± 6.92 ^##^	37.29 ± 2.51 ^##^

** *p* < 0.01, compared with group I; ^##^*p* < 0.01, compared with group II.

## 3. Discussion

Reactive oxygen species (ROS) are formed under physiological and pathological conditions in mammalian tissues. ROS play a key role in several pathogenic processes. Free radicals are produced *in vivo* during normal metabolism by enzymes such as xanthine oxidase and nitric oxide synthase and most abundantly by the electron transport chain during oxidative phosphorylation. Although transient, species such as the superoxide radical and the hydroxyl radical can overcome natural antioxidant defenses, altering proteins, nucleic acids, and lipids (lipid peroxidation). This results in cell injury or death, subsequent tissue damage and, ultimately, a chronic disease state [22]. Therefore, the generation of ROS by various reactions becomes important as it may enhance the development of a disease in which one of the factors in the etiology of the disease is oxidative stress [23]. There are complex antioxidant defense systems in organism, including both enzymatic (CAT, catalase; SOD, superoxide dismutase and GPx, glutathione peroxidase) and nonenzymatic components (such as b-carotene; retinal; α-tocopherol; ascorbic acid; reduced glutathione, GSH) against the effects of ROS [24].

MDA, an end product of membrane lipid peroxidation, is one of the most widely used markers for free radical mediated damage. Many investigators have measured lipid peroxidation levels in AOM and acute or chronic tonsillitis in both experimental [25,26,27,28,29] and human [30,31,32,33,34] studies. These studies also demonstrated that increased oxidative stress in the both diseases. Nitric oxide is a radical molecule produced by induced nitric oxide synthases (iNOS) and is produced in large amounts once iNOS is formed [35]. Nitric oxide plays a role in the inflammatory response in the middle ear. Nitric oxide and free oxygen radicals can interact in the human middle ear in a reaction sequence that eventually leads to the formation of tympanosclerosis [36]. Our results confirmed that oxidative injury was closely associated with otitis media.

In our study, antioxidant enzymes SOD, CAT, GSH-Px and GR activities in the blood and cochlea samples of OM model group (Group II) were significantly lower than those of the normal group (Group I). By contrast, administration of Ginkgo leaf parenteral solution for 15 days dose-dependently significantly enhanced the SOD, CAT, GSH-Px, GR activities in Group III and IV rats when compared to Group II rats. These results showed that antioxidant enzyme activity disorder tend to produce free radicals. Increased levels of free radicals may be responsible for the damage occurring in middle ear. Administration of Ginkgo leaf parenteral solution could decrease oxidative injury in OM rats by enhanced body antioxidant enzymes activities.

TNF-α is produced by macrophages and epithelial cells. It plays a critical role in mediating inflammatory responses by up-regulation of genes encoding cell-adhesion molecules required for the recruitment of inflammatory cells and genes encoding important inflammatory cytokines, such as IFN-γ [37]. Earlier studies have documented the presence of several cytokines, including tumor necrosis factor (TNF) α, interleukin (IL) 1β, IL-2, IL-6, and IL-8, interferon-γ, and TNF soluble receptor, in middle ear effusions in humans and experimental animals [38,39,40,41,42,43,44]. Interleukin-10 (IL-10 or IL10), also known as human cytokine synthesis inhibitory factor (CSIF), is an anti-inflammatory cytokine. In humans IL-10 is encoded by the IL10 gene [45]. IL-10 has also been shown to inhibit formation of interferon-γ and production by macrophages of IL-1, IL-6, and TNF-α, but has not been investigated in middle ear effusions. In our work, the TNF-α, IL-1β, IL-6, IL-8 and IL-10 levels were significantly increased in group II when compared with group I. However, administration of Ginkgo leaf parenteral solution for 15 days dose-dependently significantly decreased the TNF-α, IL-1β, IL-6, IL-8 and enhanced IL-10 levels in cochlea of Group III and IV rats when compared with the OM model group (II). This indicated that the TNF-α, IL-1β, IL-6, IL-8 and IL-10 involved into inflammatory response in otitis media. IL-10 is capable of inhibiting synthesis of pro-inflammatory cytokines like IFN-γ, IL-2, IL-3, TNF-α and of suppressing the antigen presentation capacity of antigen presenting cells [46]. The low level of IL-10 in OM might indicated immunity dysfunction and acute inflammation in body and may lead to persistent inflammation and irreversible organ injury. Increased IL-10 level may modulate abnormal immunity, inhibit inflammation reaction and promote restoration of inflammatory tissue. Ginkgo leaf parenteral solution produces its anti-inflammatory effects by inducing the production of IL-10, therefore, administration of Ginkgo leaf parenteral solution could decrease inflammation in rats with otitis media.

## 4. Experimental

### 4.1. Materials

Ginkgo leaf parenteral solution were purchased from Guizhou YiBai Pharmacology Ltd. (Guizhou, China).

### 4.2. Preparation of Formalin-Killed NTHI Strains

The use of whole, formalin-killed NTHi as a source of LOS was developed in the laboratory of the PLA General Hospital and has been described in detail previously [47]. NTHi strains were grown on sBHI agar, with or without antibiotics as indicated above, overnight in a CO_2_ incubator at 37 °C. The bacteria were killed by incubation with 0.3% formalin at room temperature for 24 h. Killed NTHi were washed and suspended in sterile phosphate buffered saline (PBS) as described previously [48].

### 4.3. Study Design

Twenty-four male Wistar rats (240–270 g) were randomly assigned to four cohorts and anesthetized by intramuscular injection with ketamine hydrochloride (80 mg/kg of body weight) and xylazine (8 mg/kg). OM was then induced by the direct bilateral inoculation of the middle ears, with 30 mL of a suspension containing 10^8^ CFU of formalin-inactivated NTHi in sterile PBS as previously described [49,50] and received subcutaneous injection of saline (Group II). OM was then induced by the direct bilateral inoculation of the middle ears, with 30 mL of a suspension containing 10^8^ CFU of formalin-inactivated NTHi in sterile PBS and received subcutaneous injection of ginkgo leaf parenteral solution [0.2 mL/kg (Group III), 0.4 mL/kg (Group IV), 0.6 mL/kg (Group V)]. Inoculations were made through the bony wall of the cephalid bullae, which was accessed through a neutral midline incision and blunt dissection. An additional eight rats were used as normal controls (Group I) without induction and injections. All animal experiments were approved by the Institutional Animal Care and Use Committee at the PLA General hospital. The experiment lasted for 15 days. After this time, eight rats of each group were killed by cervical decapitation under light ether anesthesia. Blood and cochlea of each rat were quickly excised, cleared of adhering fat, rinsed with a cold 0.9% sodium chloride solution, and weighted. Sample was immediately submerged in 7% perchloric acid and 2 mM phenanthroline and homogenized with a Potter-Elvejhem homogenizer at 0 °C until a uniform suspension was obtained. 

### 4.4. Biochemical Analysis

TNF-α, NO, IL-1β, IL-6, IL-8 and IL-10 levels were measured by using commercial ELISA kits. SOD activity was measured based on the ability of the enzyme to inhibit the autoxidation process of pyrogallol using a modification of the procedure described by Marklund and Marklund [51]. Briefly, the tissues were homogenized in 50 mmol/L phosphate buffer (pH 7.8) using a Polytron homogeniser. The homogenate was centrifuged at 1,600 g for 15 min. Pyrogallol solution (10 mmol/L, 2 mL) was added to various concentrations of the tissue supernatants and the rate of autoxidation was measured spectrophotometrically at 420 nm. SOD activity is expressed as units of SOD/mg protein (1.0 U is defined as the amount of the enzyme, which causes 50% inhibition of pyrogallol autoxidation).

CAT activity was measured in 10% liver homogenate prepared in phosphate buffer, centrifuged at 9,000 g, 4 °C for 15 min. The activity was determinated as described by Aebi [52]. Protein concentration was determined according to Lowry *et al*. [53], using bovine serum as a standard. Enzyme activities were expressed as units of enzyme activity per miligrams of protein.

MDA concentration was determined as a thiobarbituric acid reactive substances in the liver homogenates in 0.15 M KCl according to Buege and Aust [54].

The method of Lawrence and Burk [55] as described by Mantha *et al*. [56] was used to measure GSH-Px activity. The assay mixture consisted of 75 mmolar phosphate buffer (pH 7.0, 2.0 mL), 60 mmolar glutathione (50 μL), glutathione reductase (0.1 mL, 30 units/mL), disodium EDTA (0.1 mL, 15 mmolar), reduced NADPH (0.1 mL, 3 mmolar) and an appropriate amount of tissue supernatant to a final volume of 3.0 mL. The reaction was started by addition of 7.5 mmolar H_2_O_2_ (0.1 mL). The rate of change of absorbance during the conversion of NADPH to NADP^+^ was recorded spectro-photometerically at 340 nm for 3 min. GSH-Px activity was expressed as lmol of NADPH oxidized to NADP^+^ min^−1^ mg^−1^ protein using an extinction coefficient (6.22 mM^−1^ cm^−1^) for NADPH. Protein content of the samples was determined by the Biuret method of Gornall *et al*. [57].

GR Activity was determined by following the oxidation of NADPH to NADP^+^ during the reduction of oxidized glutathione (GSSG) [58]. The main reagent was prepared by combining KH_2_PO_4_ buffer (18.00 mL, 139 mmol/L, 0.76 mmol/L EDTA; pH 7.4) and NADPH (2.5 mmol/L, 2.00 mL). The sample (20 µL of 1:20 hemolysate plus 20 µL of KH_2_PO_4_ buffer), the main reagent (220 µL), and FAD (315 µmol/L, 5 µL) plus KH_2_PO_4_ buffer (10 µL) were added to the cuvette, and the absorbance at 340 nm was monitored for 200 s (step A). Then GSSG (22 mmol/L, 30 μL; Sigma G-4376) plus KH_2_PO_4_ buffer (10 µL) were added to start the reaction and the absorbance was followed for 175 s. The final reaction volume was 315 µL. The difference in absorbance per minute between steps B and A was used to calculate the enzyme activity by using a molar absorptivity of NADPH at 6.22 × 10^3^ L mol^−1^ cm^−1^. The unit is µmol of NADPH oxidized/min.

### 4.5. Statistical Analysis

All values were expressed as means ± SEM. The differences between control and ethanol-treated groups were compared by Student’s *t*-test using standard statistical packages. The results were considered significant only if the P value was less than 0.05.

## 5. Conclusions

Our work suggested that otitis media was closely associated with oxidative injury. Administration of Ginkgo leaf parenteral solution could enhance antioxidant enzyme activities, decrease oxidative injury, and subsequently alleviate inflammation in rats with otitis media.
